# Mr. George Aston Pedley

**Published:** 1911-05-20

**Authors:** 


The sudden death of Mr. George Aston Pedley is announced at Winchester. He studied at St. Mary's, Guy's, and the National Dental Hospital, and took his M.R.C.S. and L.S.A. in 1886, his L.R.C.P. in 1887, and the L.D.S. in 1897. He had been dental surgeon at Tonbridge School, surgeon to the Irrawaddy Flotilla Company, hon. surgeon to the Home for Lepers, Mandalay, surgeon-captain to the Upper Burma Volunteer Rifles, and dentist to the Alresford Union.

